# EGL-20/Wnt and MAB-5/Hox Act Sequentially to Inhibit Anterior Migration of Neuroblasts in *C*. *elegans*

**DOI:** 10.1371/journal.pone.0148658

**Published:** 2016-02-10

**Authors:** Matthew P. Josephson, Yongping Chai, Guangshuo Ou, Erik A. Lundquist

**Affiliations:** 1 Programs in Genetics and Molecular, Cellular and Developmental Biology, Department of Molecular Biosciences, University of Kansas, Lawrence, KS, 66045, United States of America; 2 School of Life Sciences, Tsinghua University, Beijing, 100084, China; Laboratoire de Biologie du Développement de Villefranche-sur-Mer, FRANCE

## Abstract

Directed neuroblast and neuronal migration is important in the proper development of nervous systems. In *C*. *elegans* the bilateral Q neuroblasts QR (on the right) and QL (on the left) undergo an identical pattern of cell division and differentiation but migrate in opposite directions (QR and descendants anteriorly and QL and descendants posteriorly). EGL-20/Wnt, via canonical Wnt signaling, drives the expression of MAB-5/Hox in QL but not QR. MAB-5 acts as a determinant of posterior migration, and *mab-5* and *egl-20* mutants display anterior QL descendant migrations. Here we analyze the behaviors of QR and QL descendants as they begin their anterior and posterior migrations, and the effects of EGL-20 and MAB-5 on these behaviors. The anterior and posterior daughters of QR (QR.a/p) after the first division immediately polarize and begin anterior migration, whereas QL.a/p remain rounded and non-migratory. After ~1 hour, QL.a migrates posteriorly over QL.p. We find that in *egl-20/Wnt*, *bar-1/β-catenin*, and *mab-5/Hox* mutants, QL.a/p polarize and migrate anteriorly, indicating that these molecules normally inhibit anterior migration of QL.a/p. In *egl-20/Wnt* mutants, QL.a/p immediately polarize and begin migration, whereas in *bar-1/β-catenin* and *mab-5/Hox*, the cells transiently retain a rounded, non-migratory morphology before anterior migration. Thus, EGL-20/Wnt mediates an acute inhibition of anterior migration independently of BAR-1/β-catenin and MAB-5/Hox, and a later, possible transcriptional response mediated by BAR-1/β-catenin and MAB-5/Hox. In addition to inhibiting anterior migration, MAB-5/Hox also cell-autonomously promotes posterior migration of QL.a (and QR.a in a *mab-5* gain-of-function).

## Introduction

The directed migration of neurons and neuroblasts is important in nervous system development to establish proper connectivity and circuits. Wnt signaling has been broadly implicated in mammalian cortical and hippocampal neurogenesis [1–3]. The Q neuroblasts in *C*. *elegans* have been a useful system in which to dissect the molecular mechanisms of directed neuroblast and neuronal migration [4]. The Q cells are bilateral neuroblasts in the posterior region of *C*. *elegans*. The left and right Q cells produce three neurons through an identical pattern of division and programmed cell death [4–6]. Despite these similarities, QR and descendants migrate to the anterior, and QL and descendants migrate to the posterior. The Q neuroblasts are born in embryogenesis as the sister cells of the V5 hypodermal seam cell and reside between the V4 and V5 seam cells in the posterior-lateral region [5, 6]. At hatching in the L1 larva, the Q neuroblasts undergo an initial protrusion and migration such that by 4h post hatching in the L1, QL has migrated posteriorly atop the V5 seam cell, and QR has migrated anteriorly atop the V4 seam cell [4, 7, 8]. After migrating, the Q neuroblasts undergo their first cell division producing anterior and posterior daughters (QR.a/p and QL.a/p). A posterior EGL-20/Wnt signal induces the expression of the MAB-5 Hox transcription factor in QL.a/p but not QR.a/p via canonical Wnt signaling and BAR-1/β-catenin [9–17]. MAB-5 is required for the further posterior migration of QL descendants. After this initial directional decision, Wnts redundantly control both QL and QR descendant migrations [18].

The QR descendant AQR and QL descendant PQR migrate the longest distances to the anterior and posterior of any of the three Q descendant neurons, with AQR residing near the anterior deirid and posterior pharyngeal bulb and PQR residing among the phasmid ganglia posterior to the anus [7]. In *egl-20* and *mab-5* loss-of-function mutants, the initial anterior and posterior migrations of QR and QL are normal [7], but QL descendants fail to migrate posteriorly and instead migrate anteriorly [19]. In a *mab-5* gain-of-function mutant, both QR and QL descendants migrate posteriorly despite normal initial migration of QR to the anterior and QL to the posterior [7, 15, 19]. Thus, MAB-5 is a determinant of posterior migration and can act in both QL and QR descendants, but normally only acts in QL due to EGL-20/Wnt-induced expression of MAB-5 in QL but not QR. The patterns of cell division and cell death, and the gross differentiation of the three neurons produced from each cell are unaffected by *mab-5* mutation, suggesting that *mab-5* is involved in terminal differentiation to specify direction of migration, and not cell fate or differentiation generally.

While it is clear that EGL-20/Wnt and MAB-5 promote posterior migration, the mechanism by which these molecules alter QL.a/p behavior relative to QR.a/p during posterior versus anterior migration remains unclear. In this study, we analyzed the behavior of the QR.a/p and QL.a/p cells as they migrate in wild-type, *egl-20*, and *mab-5* mutants. We found that in wild-type, QR.a/p began anterior migration shortly after division, whereas QL.a/p retained a rounded, non-migratory morphology. Approximately one hour after QR.a/p migration, QL.a began to migrate posteriorly over QL.p. In *mab-5* and *egl-20* loss-of-function, QL.a/p polarized and migrated anteriorly, similar to QR.a/p in wild-type, suggesting that both normally inhibit anterior QL.a/p migration. However, in *mab-5*, QL.a/p transiently retained a rounded, non-migratory morphology, whereas in *egl-20*, QL.a/p began migration shortly after division, similar to QR.a/p in wild-type. *bar-1/β-*catenin mutants resembled *mab-5* and displayed the transient non-migratory morphology not seen in *egl-20*. These results suggest that EGL-20 has both an acute MAB-5-independent role and a later MAB-5-dependent role in inhibiting anterior QL.a/p migration, and that the acute role of *egl-20* does not involve BAR-1/β-catenin. The *mab-5* gain-of-function mutant caused QR.a/p posterior migration similar to QL.a/p, including posterior migration of QR.a. Thus, in addition to inhibiting anterior migration, MAB-5 also promoted posterior migration.

## Results

### Wnt Signaling Redundantly Controls AQR and PQR Migration

**Fig 1:**
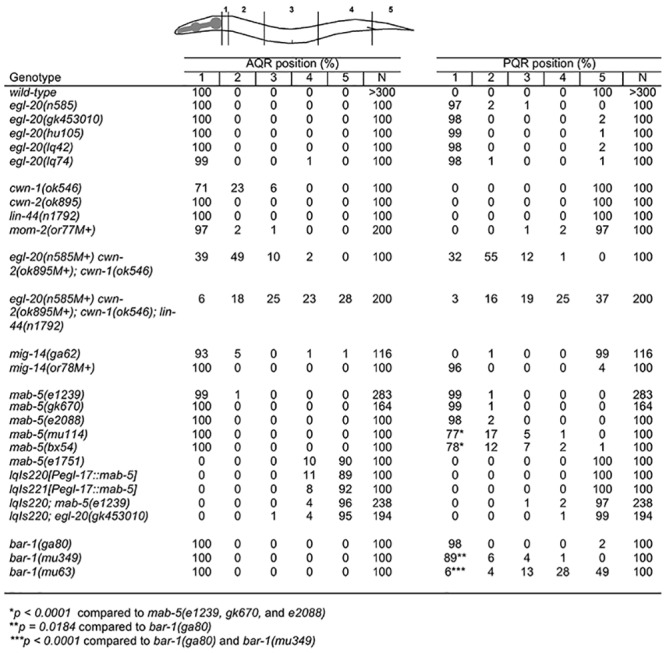
AQR and PQR migration. The percentages of AQR and PQR in mutants is shown, with respect to postions 1–5 as described in Methods and shown on the diagram. Statistical significance was calculated using Fisher’s Exact test.

A genome-wide screen for new mutations affecting AQR and PQR migration identified two new mutations, *lq42* and *lq74*. Both caused anterior PQR migration, and minor or no defects in AQR migration (Fig 1). Genes affected by the mutations were mapped by whole genome single nucleotide polymorphism resequencing using the Cloudmap protocol [20] (data not shown). Both mapped to a region in the center of LG IV, and each harbored a mutation in the *egl-20* gene, which encodes a *C*. *elegans* Wnt molecule. *egl-20* has been shown previously to affect PQR but not AQR migration [7], consistent with the phenotypes of *lq42* and *lq74*. *lq42* introduced a premature stop codon at arginine 296 (C to T), and *lq74* was a missense mutation changing cysteine 248 to tyrosine (G to A) (see Methods). Both failed to complement *egl-20(n585)* for PQR migration (data not shown). The *egl-20(gk453010)* allele was generated by the Million Mutation Project (present in strain VC40076) [21]. *gk453010* is a G to A transition resulting in an arginine 245 to opal stop codon mutation. The *egl-20(hu105)* allele also introduces a premature stop codon [22, 23]. *lq42*, *lq74*, *hu105*, and *gk453010* each displayed nearly completely penetrant anterior PQR migration (Fig 1), suggesting that they are all putative null mutations, with the exception of the *lq74* missense mutation which may retain some function (1% PQR did not migrate completely to head).

AQR and PQR migration was analyzed in mutants of the four other *C*. *elegans Wnt* genes *cwn-1*, *cwn-2*, *lin-44*, and *mom-2* (Fig 1). Putative null alleles of *cwn-2* and *lin-44* displayed no defects. A null *cwn-1* allele displayed 29% failure of AQR to complete anterior migration. *mom-2*, which has not been previously reported to affect Q descendant migrations, displayed 3% AQR and PQR migration defects in the partial loss of function *or77M+* allele (M+ is used to designate homozygous animals from a heterozygous mother and therefore with a wild-type maternal contribution of gene function). Thus, *egl-20*, *cwn-1*, and, *mom-2* each affect AQR and PQR migration in distinct ways.

Redundancy among Wnts in Q descendant migrations has been reported previously [18]. The *Wnt* triple mutant *egl-20(n585) cwn-2(ok895); cwn-1(ok546)* displayed highly penetrant PQR anterior migration and a failure of both AQR and PQR migration along the anterior route (Fig 1). No AQR or PQR migrated posteriorly in this genotype. Mutation of *lin-44* in the *egl-20(n585M+) cwn-2(ok895M+); cwn-1(ok546); lin-44(n1792)* quadruple mutant resulted in posterior migration of both AQR and PQR (28% and 37%) (Fig 1). This indicates that *lin-44* activity inhibits posterior AQR and PQR migration in the absence of *egl-20*, *cwn-1*, and *cwn-2*, and suggests that *lin-44* can act as a posterior repellant. Previous studies indicated that *lin-44* caused posterior displacement of QL descendants PVM and SDQL [18], and HSN [24], in certain multiple Wnt mutant combinations, consistent with a posterior repellant activity of *lin-44*. The triple and quadruple Wnt mutants were scored with maternal contribution of both *egl-20* and *cwn-2*, so we cannot be certain that maternal Wnt function remains in these mutants. MIG-14/Wntless is required for Wnt processing and function and affects QL descendant migrations [25]. Two *mig-14* mutants had less severe defects than the *Wnt* triple and quadruple mutants (Fig 1). The likely hypomorph *ga62* had weak effects on both AQR and PQR, and the lethal in-frame deletion mutation *or78*, with wild-type maternal contribution, resembled *egl-20* and only affected PQR migration. These data suggest either that these mutations did not completely eliminate *mig-14* function, or that *mig-14* is not involved in all Wnt-related signaling events.

### Wnt Signaling Might Not Affect Early Q Neuroblast Migration

The above results, along with previous studies [18, 26], indicate that the five *C*. *elegans Wnt* genes and four *Frizzled* genes *mig-1*, *lin-17*, *cfz-2*, and *mom-5* act redundantly to control Q descendant migration. However, *egl-20* alone and the *egl-20(n585) cwn-2; cwn-1* triple mutant displayed no defects in early Q anterior-posterior protrusion and migration, despite severe AQR and PQR descendant migration defects [7, 26] (Fig 1). To further investigate the role of Wnts in early Q protrusion and migration, we analyzed early QL and QR protrusion and migration with the *ayIs9[Pegl-17*::*gfp]* transgene, expressed in the early Q cells [27–29]. Two additional mutant combinations were tested: the *Wnt* triple mutant *egl-20(lq42M+); mom-2(or77M+); lin-44(n1792)* and the *Frizzled* double mutant *mom-5(ne12M+) lin-17(n761M+)*. Neither displayed defects in direction of initial QL and QR migration (QL divided atop V5 and QR atop V4 (n ≥ 10)) (data not shown). We have not scored early Q migrations in the absence of all Wnt signaling due to maternal perdurance and/or incomplete knockdown of all Wnt genes at once. However, we have not observed early Q directional migration defects in any Wnt signaling mutant combination tested, despite strong AQR and PQR migration defects. This suggests Wnt signaling might not be involved in early Q anterior-posterior protrusion and migration, with function restricted to later migrations of the Q descendants.

### QR Daughters Begin Migration Immediately after QR Division

**Fig 2:**
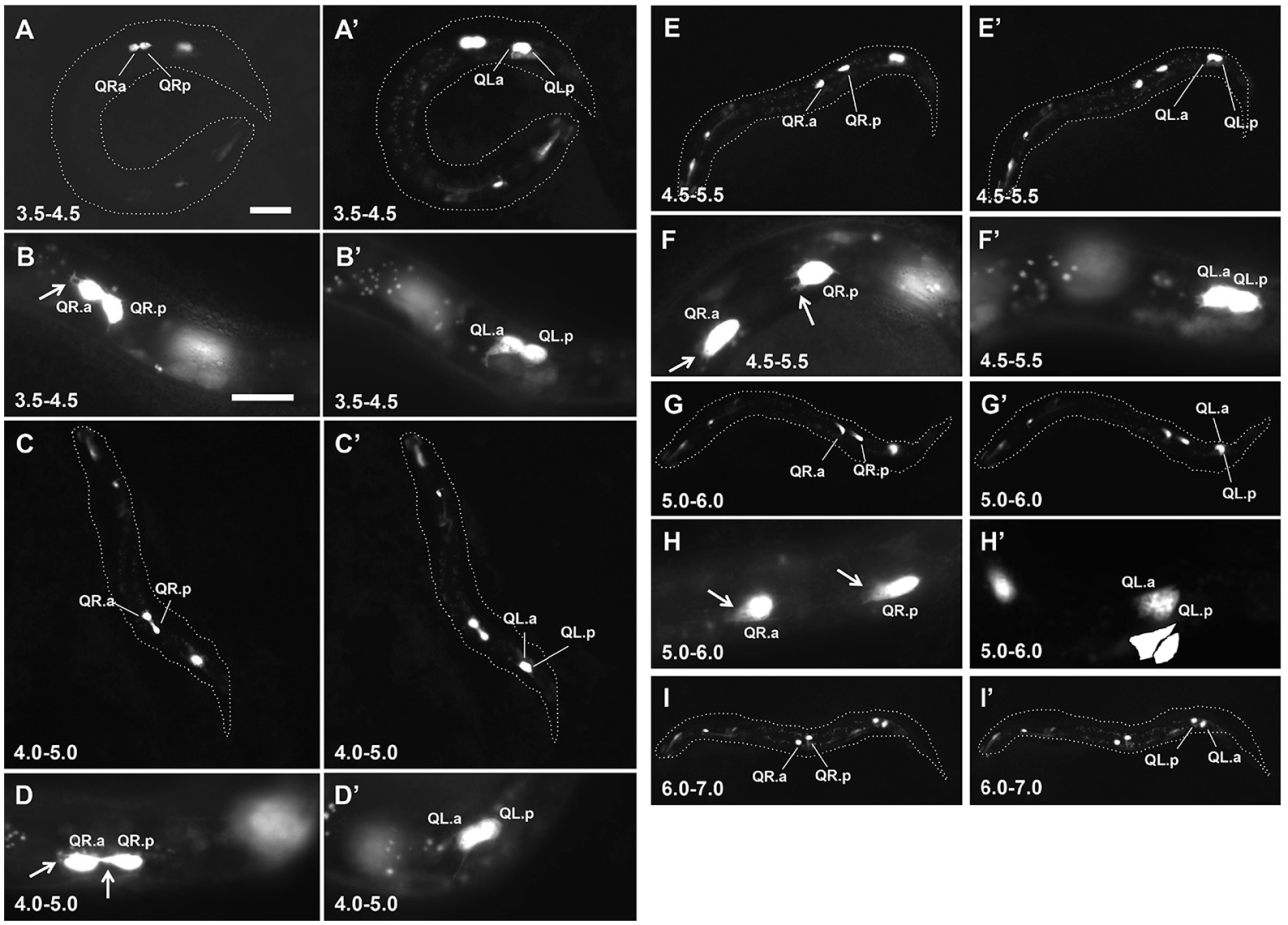
Q descendant migrations in *wild-type*. Fluorescent micrographs of *ayIs9[Pegl-17*::*gfp]* expression in Q descendants are shown in the same animals (QR, A-I; QL, A’-I’). Anterior is to the left. The dashed lines indicate the body of the whole animal in the micrograph. Arrows indicate anterior protrusions characteristic of the QR migratory morphology. The scale bar in A represents 5μm for A, C, E, G, and I; and the scale bar in B represents 5μm for B, D, F, and H.

In order to understand how EGL-20/Wnt signaling drives posterior QL descendant migration, the behaviors of QR.a/p and QL.a/p just after division were analyzed. We monitored the position of the Q cell daughters using a *Pegl-17*::*gfp* transgene *ayIs9* expressed in the Q cells (Fig 2). Newly-hatched L1 larvae were synchronized within the same hour-long age range at half-hour timepoints after hatching (e.g. 3.5–4.5h, 4.0–5.0h, 4.5–5.5h, 5.0–6.0h, 5.5–6.5h, 6.0–7.0h) (see Methods for synchronization of L1 larvae). The positions of QR, QL, and their daughters were determined in 20 animals at each timepoint. At 3.5–4.5h, 19/20 of the QR examined had divided, whereas only 13/20 of the QL had divided. This trend held true in all genotypes analyzed, suggesting that QL divides slightly later than QR. Indeed, at 4.0–5.0h, 4/20 QL had not yet divided, but 20/20 of the QRs had divided.

**Fig 3:**
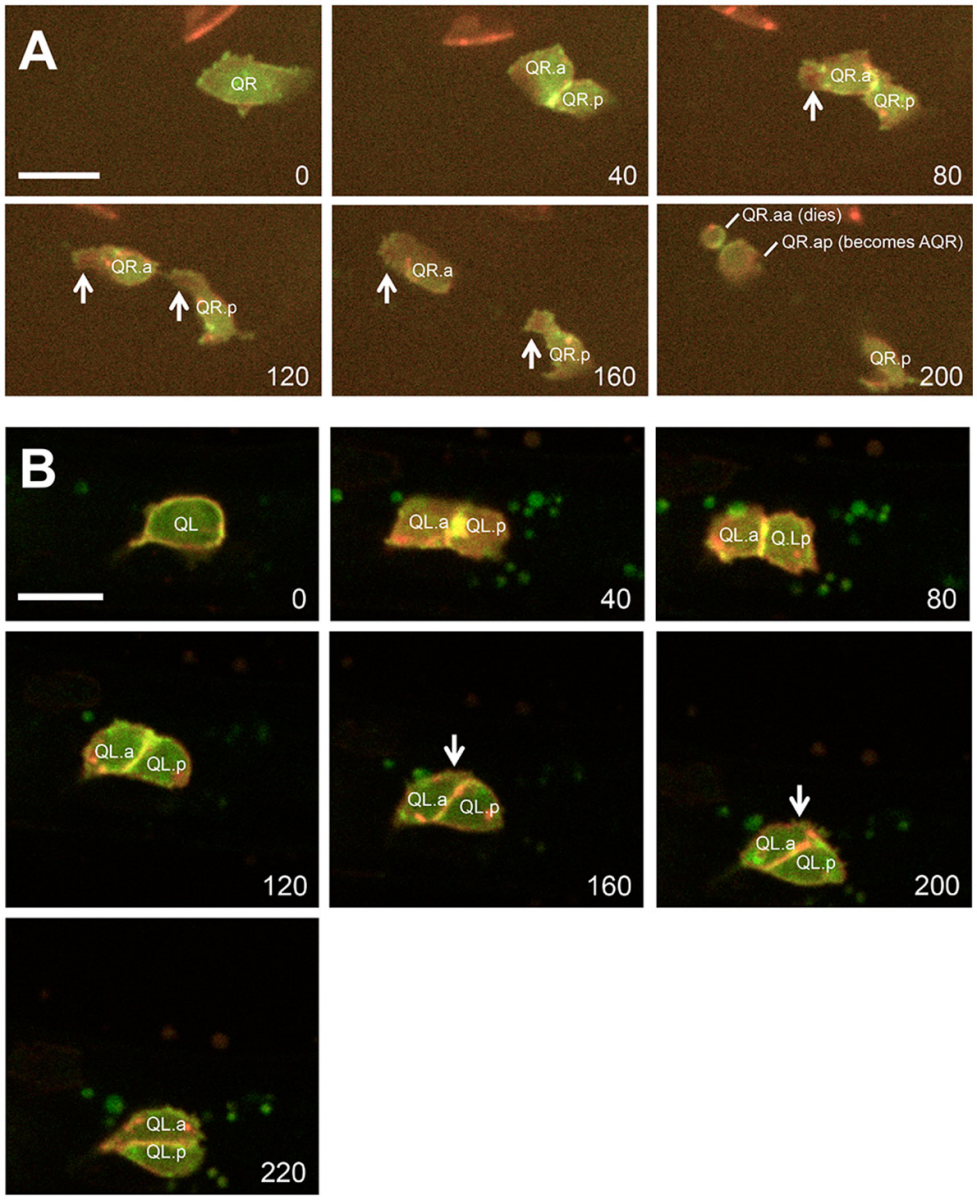
Time-lapse imaging of QR and QL descendant migrations. Micrographs from time lapse imaging of QR (A) and QL (B) are shown, using the *casIs330* transgene [31] (see Methods). Individual images were taken from S1 and S2 Movies at the timepoints in minutes indicated in the lower right of each micrograph. Red indicates MYR::mCherry at membranes and in chromatin (HIS-24::mCherry), and green represents F-actin (MOEabd::GFP). (A) QR and descendants are indicated. Arrows point to the F-actin-rich lamellipodial protrusions that accompany polarization and anterior migration. F-actin also accumulated at the furrows between dividing cells (e.g. between QR.a and QR.p at 40min, and between QR.aa and QR.ap at 200min). (B) QL and descendant migration. Arrows point to the extensions from QL.a posteriorly over QL.p. In these time-lapse imaging experiments with the *casIs330* transgene, migrations and cell divisions were delayed by ~2-fold compared to *ayIs9* still images, but the same pattern of QL.a/p and QR.a/p migration as observed with *ayIs9* still images was conserved.

After division, the QR and QL daughters had a rounded morphology with little or no protrusion (Figs 2A, 2B and 3). Shortly after division in the 4.0–5.0h timepoint, the QR daughters polarized and begun migrating to the anterior (Fig 2C and 2D). Migrating QR.a/p cells first elongated in the anterior-posterior axis and then extended F-actin-rich lamellipodia-like protrusions in the direction of migration (to the anterior), similar to what has been previously reported (Fig 3A and S1 Movie) [30–32]. The QR.a/p cells maintained this polarized, migratory morphology as they migrated anteriorly until their second round of cell division at approximately 7h post-hatching (Figs 2D–2I and 3A). At 6.0–7.0, QR.a/p cells began to lose the polarized morphology and assumed a rounded morphology in preparation for the second round of cell division (Figs 2I and 3A). Time-lapse imaging with the *casIs330* transgene (S1 Movie, S2 Movie, and Fig 3) [30] resulted in an approximately 2-fold delay in migration and division relative to the *ayIs9* transgene.

**Fig 4:**
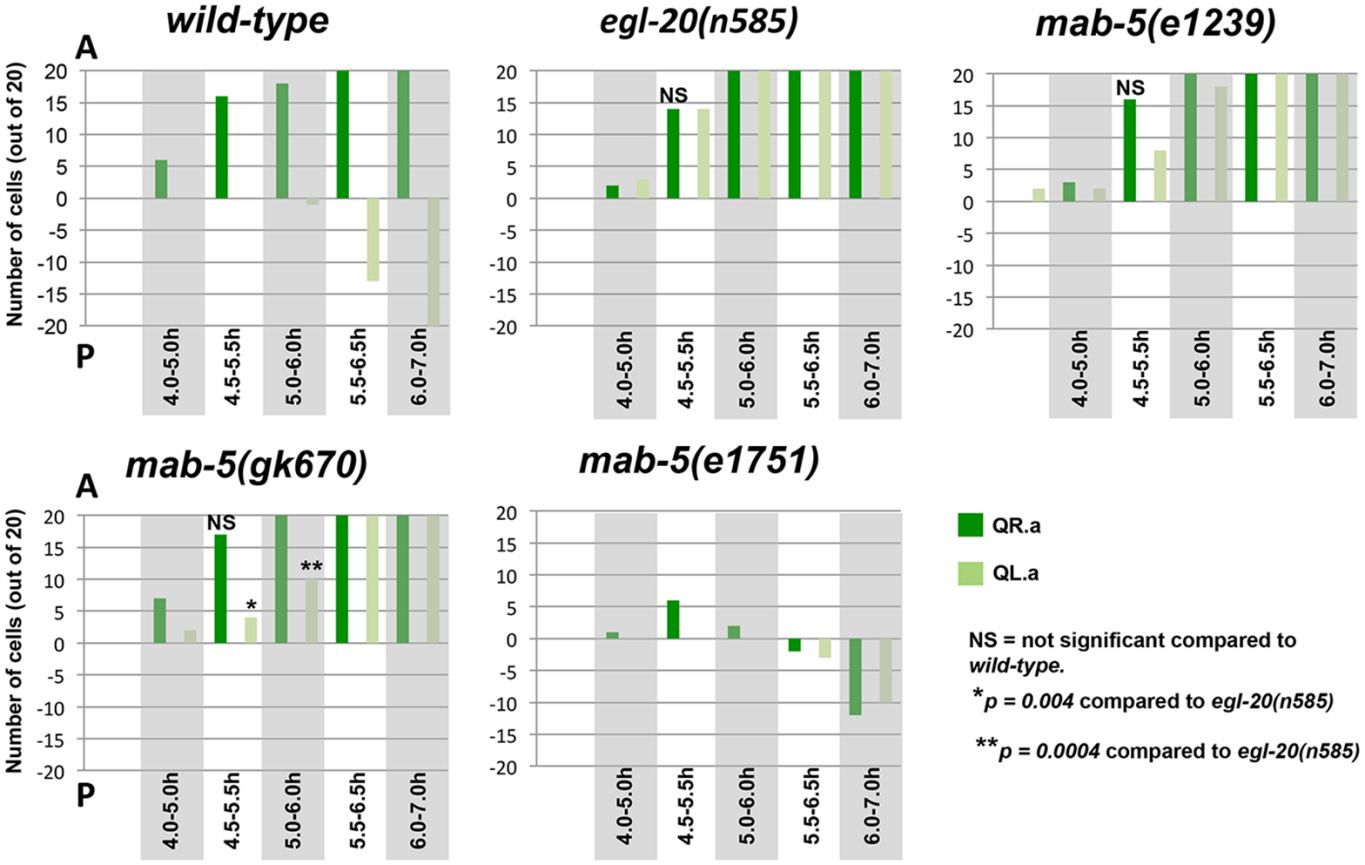
Quantitative representation of Q descendant migrations. Graphs represent Q migrations in indicated genotypes. The number (out of 20) of QR.a (dark green) and QL.a (light green) that had migrated (Y axis) at each timepoint (X axis) are indicated. Positive numbers on the Y axis represent anterior migration, and negative numbers represent posterior migration. A cell was scored a migratory if it extended an anterior lamellipodial like protrusion and separated from its sister (for anterior migration) or if it extended a posterior protrusion over its sister or migrated behind its sister (for posterior migration).

To account for variability in the timing of migration, QR.a/p migration was quantified in 20 animals at each time point using *ayIs9* (Fig 4). Cells were scored as migratory if they extended protrusions to the anterior, often accompanied by migration from their birthplace and also separation from one another (Figs 2E, 2F and 3A). At 4.0–5.0h, 6/20 of the QR daughters had polarized and started migrating to the anterior (Fig 4). At 4.5–5.5h, 16/20 were migrating, and at 5.0–6.0, 18/20. By 5.5–6.5h, all (20/20) of the QR.a/p were polarized and migrating to the anterior (Fig 4).

### QL Daughters Remain Rounded and Non-Migratory

In contrast to QR.a/p, the QL.a/p daughters did not polarize and migrate anteriorly. From 3.5–5.5h, at a time when QR.a/p had polarized and begun migration, the QL.a/p cells retained a rounded, non-migratory morphology and did not migrate (Figs 2A’–2F’ and 3B). Occasionally, protrusions were observed on non-migratory QL.a/p (e.g. Fig 2F’), but they did not assume the elongated morphology and robust anterior protrusion observed in QR.a/p. At 5.0–6.0h, the anterior QL.a daughters began migrating posteriorly over the anterior QL.p daughters, which retained a rounded morphology (Figs 2G’–2H’ and 3B) (S1 Movie). QL.p showed no significant migration over this time period, consistent with previous observations [33]. Often, QL.a apparently migrated over the top of QL.p (Figs 2H’ and 3B). QL.a was scored as migrating posteriorly if it was over QL.p, or if it was indistinguishable from QL.p suggesting it was migrating in front of or behind QL.p (data not shown).

Quantification of migration in 20 animals showed that only 1/20 QL.a began posterior migration at the 5.0–6.0h timepoint (Fig 4). At 5.5–6.5h, 13/20 of the anterior QL.a cells had migrated. At 6.0–7.0h, all QL.a had migrated posterior to QL.p and began to assume a rounded morphology preceding the second round of cell division (Fig 2I’). Posterior QL.p showed little or no polarization or migration in the entire time before the second round of cell division (7.0–8.0h after hatching).

**Fig 5:**
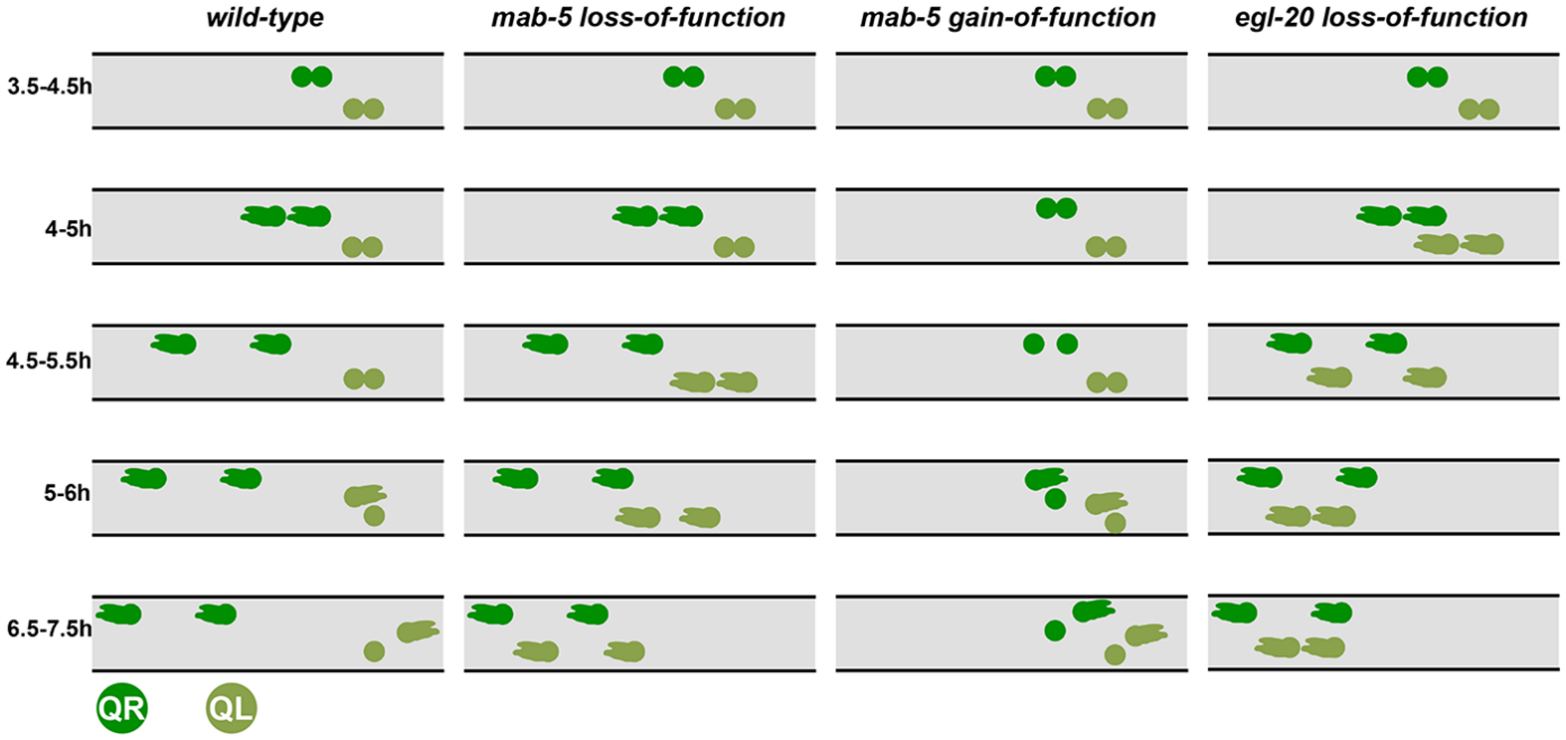
Schematic summary QR.a/p and QL.a/p migration behavior. QR.a/p are dark green and QL.a/p are light green. Genotypes and time points using *ayIs9[Pegl-17*::*gfp]* are indicated. QL.a/p begin anterior migration soon after division in the 4.0–5.0h timepoint, whereas QL.a/p in *mab-5* delay anterior migration until the 4.5–5.5h timepoint. Anterior is to the left.

In sum, QR.a/p polarized and began to migrate anteriorly shortly after division. The division of QL was delayed compared to QR, and the QL.a/p daughters remained rounded and non-migratory in the 3.5–5.0h time window while the QR.a/p daughters migrated to the anterior. At 5.0–6.0h, QL.a began migrating posteriorly over QL.p and continued posterior migration. QL.p did not migrate in this time period. A schematic summary of QR.a/p and QL.a/p migratory behavior is shown in Fig 5.

### EGL-20/Wnt and MAB-5/Hox Inhibit QL.a/p Anterior Polarization and Migration

*egl-20* and *mab-5* loss-of-function mutations caused anterior migration of PQR and did not affect AQR migration (Fig 1). *mab-5(e1239*, *gk670*, and *e2088)* all showed nearly completely penetrant PQR anterior migration, suggesting that they are all strong loss-of-function alleles. *mab-5(e2088)* was a complex rearrangement affecting the second exon of *mab-5* (see Methods). *mab-5(bx54)*, a missense mutation in the homeodomain of *mab-5* [34], and *mab-5(mu114)*, a premature stop codon early in the first exon (see Methods), displayed weaker defects, indicating that they are hypomorphic alleles.

**Fig 6:**
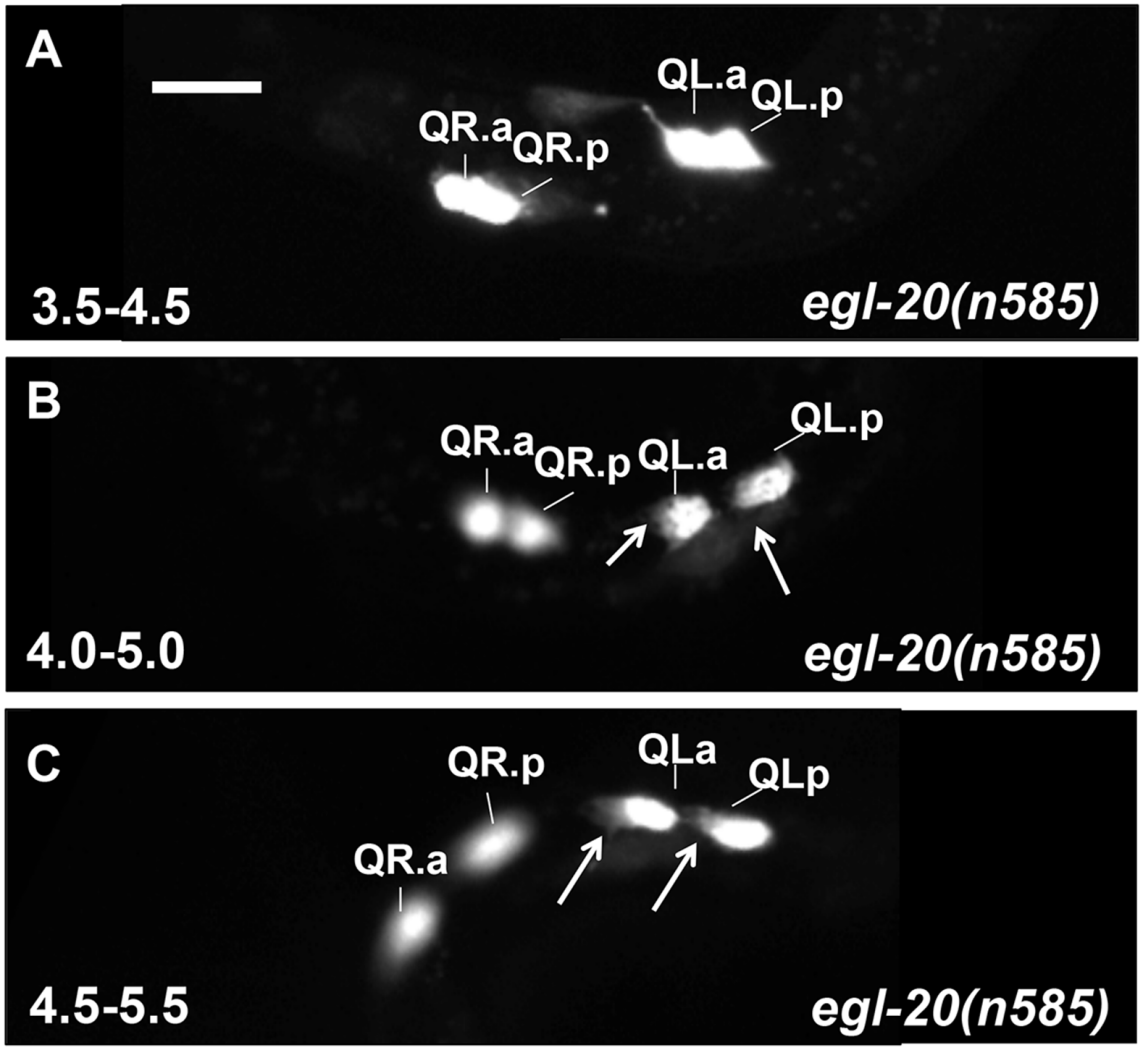
Q descendant migrations in *egl-20(n585)* mutants. Fluorescent micrographs of *ayIs9[Pegl-17*::*gfp]* expression in Q descendants are shown. Anterior is to the left. Arrows indicate anterior protrusions characteristic of migratory morphology. QL.a has begun anterior migration before QR.a in this animal at the 4.0–5.0h timepoint. The scale bar in A represents 5μm.

**Fig 7:**
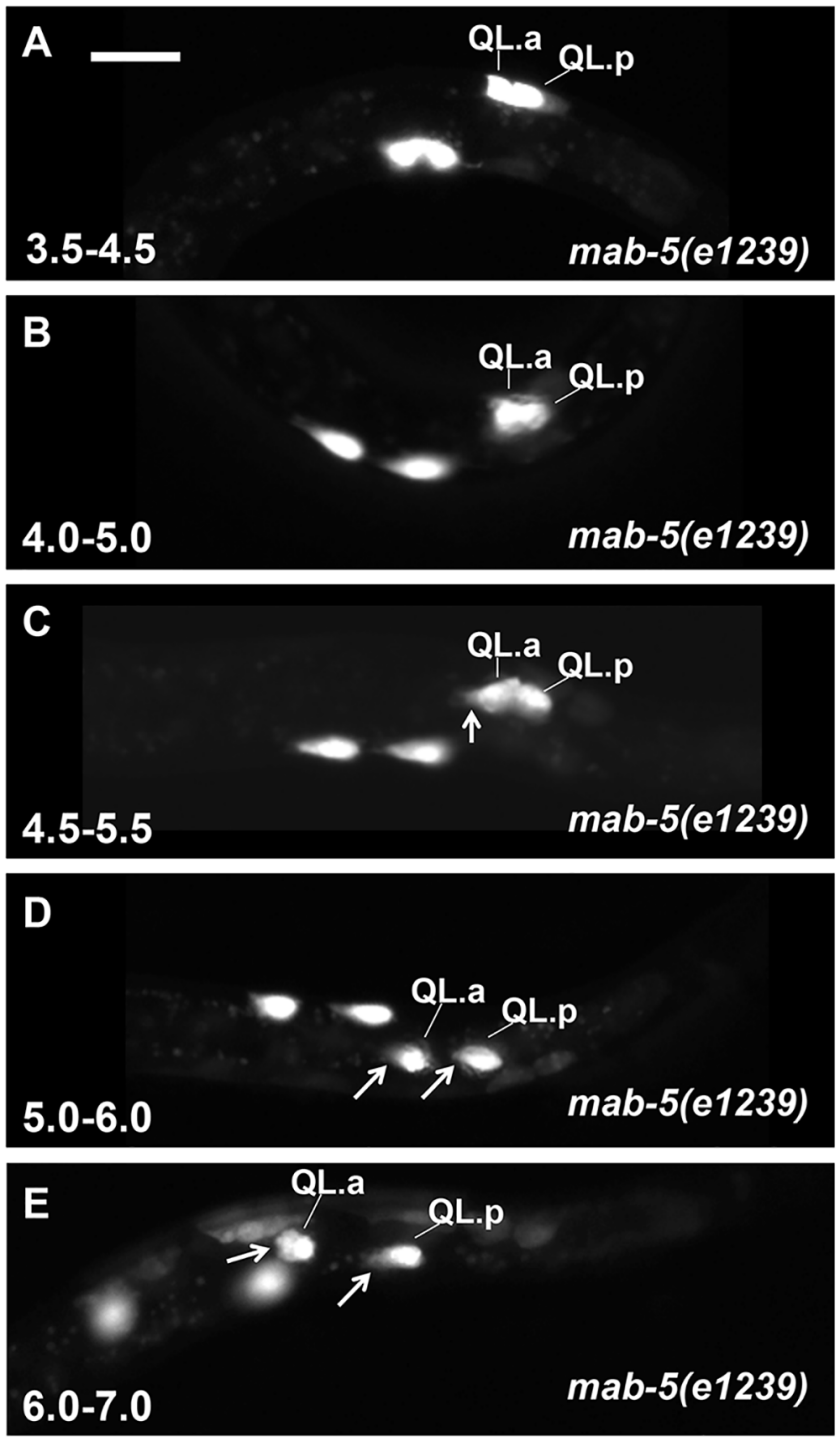
Q descendant migrations in *mab-5* loss-of-function. Fluorescent micrographs of *ayIs9[Pegl-17*::*gfp]* expression in the Q cells of *mab-5(e1239)* loss-of-function mutants are shown. At 4.0–5.0h and 4.5–5.5h, QL.a/p have not migrated, but QL.a is beginning to extend an anterior protrusion (arrow in C). Beginning at the 5.5–6.5h timepoint, both QL.a and QR.a begin posterior migration. Anterior is to the left, and the scale bar in A represents 5μm.

As expected, direction and extent of initial QR and QL migration prior to the first Q cell division was unaffected by *egl-20* and *mab-5* (Figs 4, 6A and 7A). Furthermore, the QR.a/p anterior and posterior daughters polarized and migrated to the anterior in all cases (Figs 4, 6 and 7). Previous work showed that in *egl-20* mutants, the posterior QR.p daughter displayed variable posterior polarization during migration [35]. We did not follow this phenotype in these studies.

In *egl-20(n585)*, QL.a/p polarized and migrated anteriorly shortly after division at the 4.0–5.0h timepoint, similar to QR.a/p (Fig 4). In some cases, QL.a/p polarized and began to migrate before QR.a/p (Fig 6B). Upon quantification, 3/20 QL.a/p at the 4.0–5.0 timepoint and 14/20 at the 4.5–5.5h timepoint had polarized and begun anterior migration, similar to QR.a/p (Fig 4). QL.a/p resembled QR.a/p in their anterior migrations through the remaining timepoints (Fig 6C). Thus, in the absence of EGL-20/Wnt, QL.a/p polarized and migrated anteriorly, indicating that EGL-20 inhibits polarization and anterior migration of QL.a/p.

In *mab-5* mutants, QL.a/p also polarized and migrated to the anterior similar to *egl-20* (Fig 7). Upon quantification of two *mab-5* mutants, 2/20 QL.a/p polarized and began migrating to the anterior at the 4.0–5.0h timepoint in both mutants, and 7/20 and 4/20 at the 4.5–5.5h timepoint (Fig 4). By 5.5–6.5h, all QL.a/p had migrated anteriorly in both *mab-5* mutants (Fig 4). These results suggest that MAB-5/Hox also inhibits QL.a/p anterior polarization and migration.

### *mab-5* Gain-Of-Function Causes Posterior QR.a/p Migration

**Fig 8:**
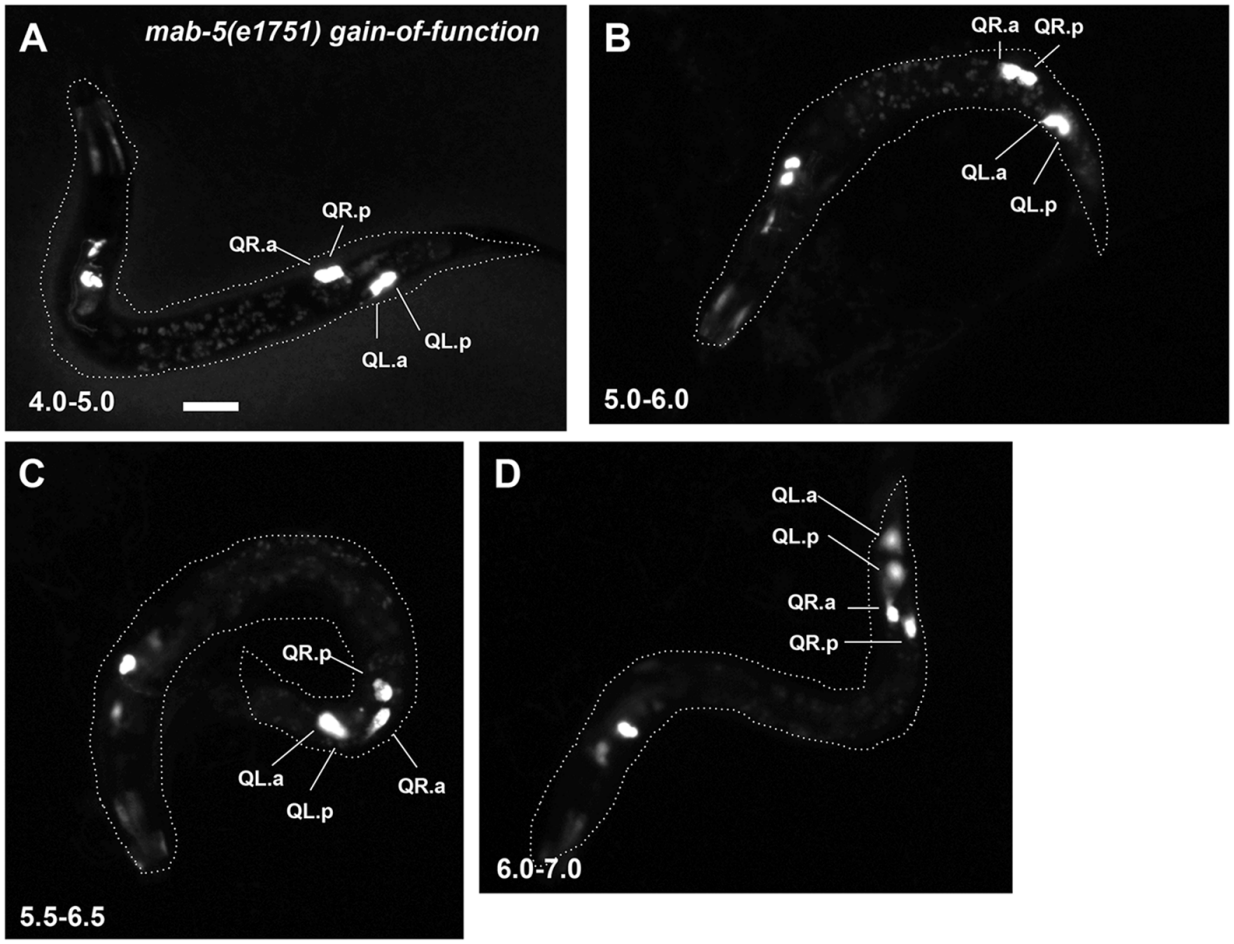
Q descendant migrations in *mab-5(e1751)* gain-of-function. Fluorescent micrographs of *ayIs9[Pegl-17*::*gfp]* expression in Q descendants are shown. Anterior is to the left. The dashed lines indicate the body of the whole animal in the micrograph. Both QL.a/p and QR.a/p retain a rounded, non-migratory morphology until 5.5–6.5h, when QL.a and QR.a begin posterior migration. At 6.0–7.0h, both anterior daughters have migrated posteriorly to the posterior daughters. The scale bar in A represents 5μm.

*mab-5(e1751)* is a gain-of-function variant that results in ectopic expression of *mab-5* in cells, including in QRs which normally do not express it [36]. *mab-5(e1751)* mutants display posterior migration of both QL and QR descendant neurons AQR and PQR, the opposite of *mab-5* loss-of-function [19] (Fig 1). We analyzed Q descendant behaviors in *mab-5(e1751)*. Initial Q migrations were normal, as QR migrated anteriorly and QL posteriorly, indicating that *mab-5(e1751)* does not affect initial Q migration (Fig 8A). QL.a/p behavior generally resembled what is seen in wild type (persistent rounded and non-migratory morphology until 5.0–6.0h, with QL.a beginning to migrate posteriorly over QL.p at this time) (Fig 8).

Strikingly, QR.a/p behaved similarly to QL.a/p in *mab-5(e1751)* (Fig 8). Some QR.a/p slightly separated from one another, but did not show the polarized and anterior migratory behavior seen in wild-type QR.a/p from 4h onward. Eventually, QR.a migrated posteriorly over QR.p just as QL.a did, beginning at the 5.5–6.5h timepoint (Figs 4 and 8). *mab-5(e1751)* resulted in a delay in QR.a/p and QL.a/p posterior migration relative to *wild-type* QL.a/p (Fig 4). At the 6.0–7.0h timepoint, all of QL.a had migrated posteriorly in *wild-type* where only 12/20 QR.a and 10/20 QL.a had migrated posteriorly in *mab-5(e1751)*. Together with *mab-5* loss-of-function, these data suggest that MAB-5 normally inhibits anterior migration of QL.a/p and can also inhibit QR.a/p anterior migration in the gain-of-function mutant (Fig 5). In addition to inhibiting anterior migration, *mab-5* activity can also induce posterior migration of the QR.a cell, similar to wild-type QL.a.

**Fig 9:**
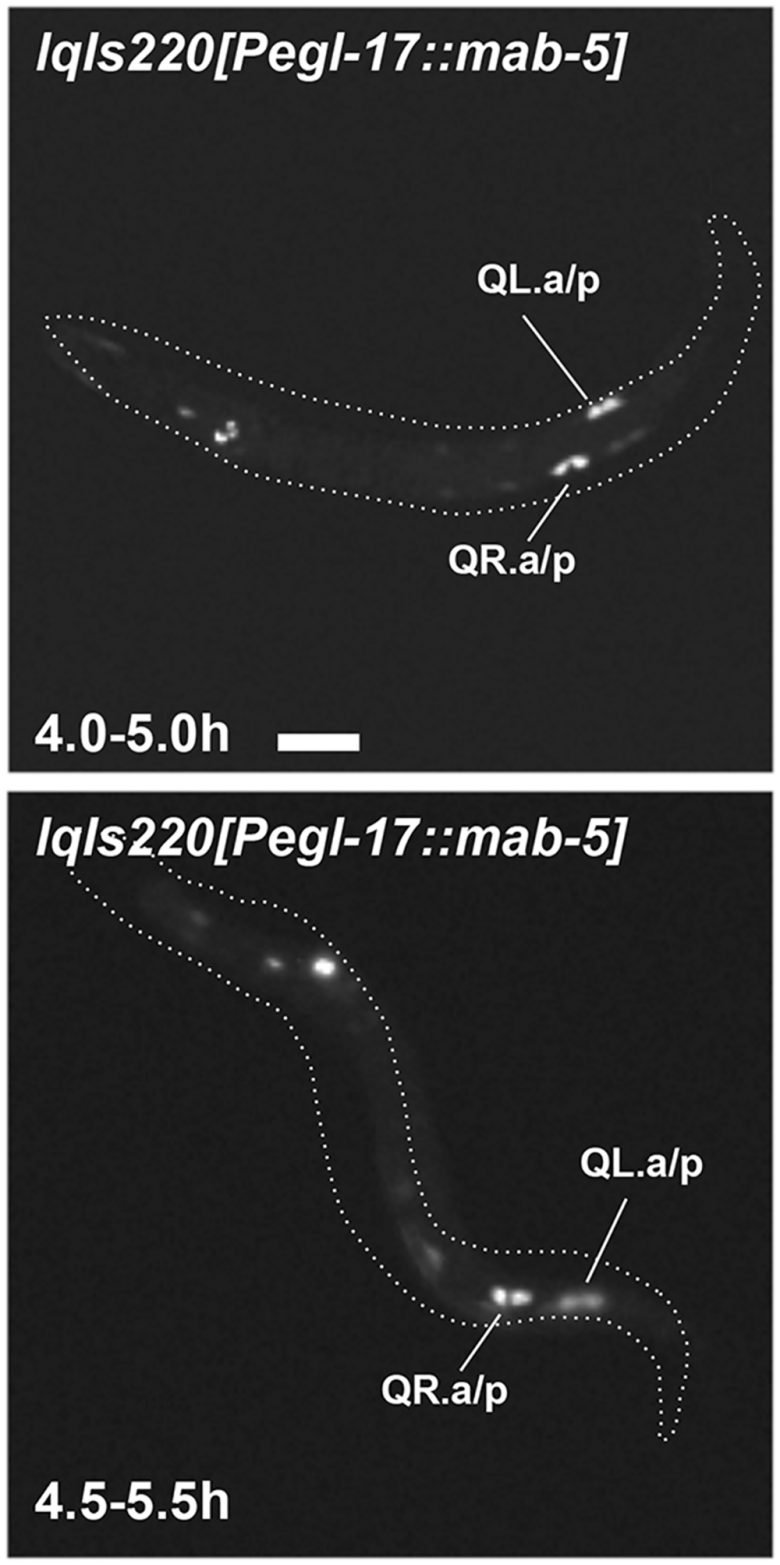
Q descendant migration in animals with transgenic *mab-5* expression in QL and QR. Fluorescent micrographs of *Pegl-17*::*gfp* expression in Q descendants are shown. Anterior is to the left. The dashed lines indicate the body of the whole animal in the micrograph. Both QR.a/p and QL.a/p are non migratory at the 4.5–5.5h timepoint. The scale bar represents 5μm for both micrographs.

*mab-5* is expressed in QL as well as in other cells surrounding QL in the posterior of the animal. We constructed a transgene driving *mab-5* expression from the *egl-17* promoter expressed in the early Q cells. Q descendant neurons AQR and PQR both migrated posteriorly in animals harboring this transgene, similar to *mab-5(e1751)* (Fig 1). Initial QL and QR migration were normal in *Pegl-17*::*mab-5* animals, but both QL.a/p and QR.a/p resembled *mab-5(e1751)* (they remained rounded and non-migratory) (Fig 9). These data indicate that MAB-5 expression in the Q cells themselves is sufficient to prevent anterior migration of QL.a/p and QR.a/p and drive their posterior migration, consistent with previous results showing autonomy of *mab-5* function in neuronal descendant migrations [15].

In *egl-20(n585); mab-5(e1751)* and *egl-20(n585); Pegl-17*::*mab-5* animals, AQR and PQR both migrated posteriorly similar to *mab-5* gain-of-function alone, consistent with *mab-5* acting downstream of *egl-20*. At the 4.5–5.5h timepoint, QL.a/p and QR.a/p resembled *mab-5(e1751)* alone and had not begun migration to the anterior as in *egl-20(n585)* (data not shown). However, both QL.a/p and QR.a/p showed the transient separation before posterior migration as observed for QR.a/p in *mab-5(e1751)* (2/21 QL.a/p and 4/21 QR.a/p for *egl-20(n585); mab-5(e1751)*, and 1/22 QL.a/p and 7/22 QR.a/p for *egl-20(n585); Pegl-17*::*mab-5*). In sum, these results indicate that the effects of *mab-5* gain-of-function are independent of *egl-20*, and are consistent with MAB-5 acting downstream of EGL-20.

### QL.a/p Anterior Migration Is Delayed in *mab-5* Mutants Compared to *egl-20*

Loss of function of *egl-20* or *mab-5* resulted in anterior migration of QL.a/p. While similar to *egl-20*, in *mab-5* mutants we noted a delay in QL.a/p polarization relative to QR.a/p not seen in *egl-20* (Fig 4). QL.a/p often remained unpolarized while QR.a/p had begun migration (Fig 7B). In *mab-5(e1239)* at 4.5–5.5h, 7/20 QL.a/p had begun migration while 16/20 QR.a/p had (Fig 4). The difference was more extreme in *mab-5(gk670)* (4/20 versus 17/20). Meanwhile, 14/20 QL.a/p began migration in *egl-20(n585)* at the same timepoint (significantly more than *mab-5(gk670)* (*p = 0*.*004*)). This delay is also observed in the 5.0–6.0h timepoint (Fig 4), where 20/20 QL.a/p began anterior migration in *egl-20(n585)*, compared to 17/20 and 10/20 (*p = 0*.*0004*) in two *mab-5* mutants, despite all QR.a/p having begun migration in *mab-5* mutants.

**Fig 10:**
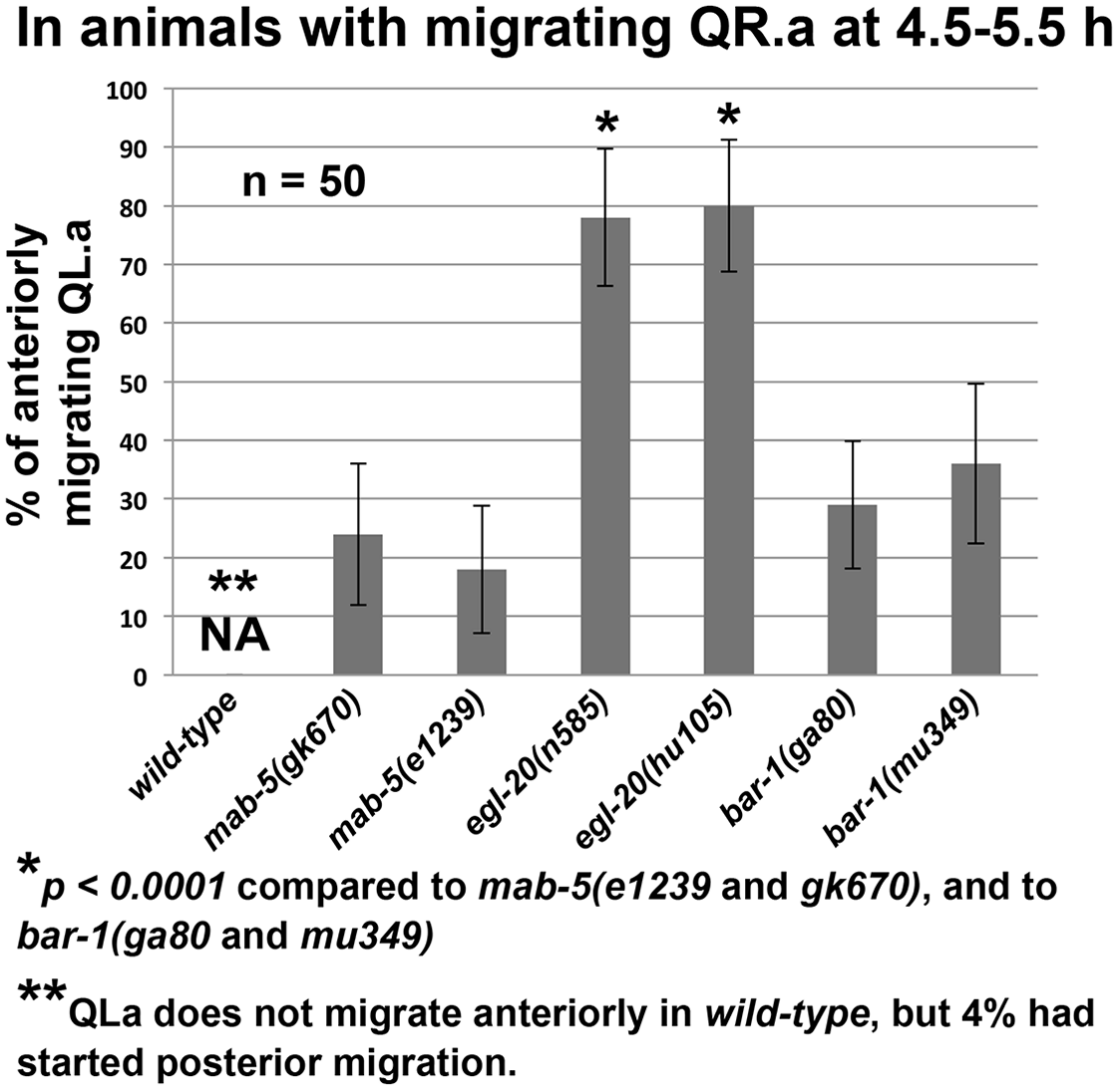
QL.a/p migrate anteriorly sooner in *egl-20* than in *mab-5*. Animals from the 4.5–5.5h timepoint with migrating QR.a were analyzed for QL.a anterior migration. The percentage of animals with anteriorly-migrating QL.a (Y axis) is indicated for each genotype (X axis). In wild-type, QL.a migrated posteriorly (4%). Error bars represent 2x standard error of the proportion, and *p* values were determined using Fisher’s exact test.

To explore this difference between *mab-5* and *egl-20* further, we determined, at the 4.5–5.5h timepoint, the proportion of QL.a/p that had polarized and begun to migrate anteriorly in animals with migrating QR.a/p (Fig 10). In wild type, 4% of QL.a had begun migration (to the posterior), while in *egl-20(n585)* and *egl-20(hu105)*, 78% and 80% of QL.a/p had begun anterior migration (*p < 0*.*05*). Furthermore, we noted that 8% of QL.a/p polarized and migrated before QR.a/p in *egl-20(n585)* (Fig 6B).

In this assay, two *mab-*5 mutants displayed significantly less QL.a/p migration compared *egl-20* (24% and 18% compared to 78% and 80%, *p < 0*.*05*) (Fig 10). These data demonstrate that QL.a/p anterior polarization and migration in *mab-5* mutants is delayed relative to *egl-20*, with QL.a/p in *mab-5* transiently retaining a rounded, non-migratory morphology (Fig 7B), a delay not observed in *egl-20* mutants.

### *bar-1/β-catenin* QL.a/p Migration Resembles *mab-5*

MAB-5 expression in QL.a/p is induced by canonical Wnt signaling involving EGL-20/Wnt and BAR-1/β-catenin [9–16]. *bar-1* loss-of-function mutations displayed anterior PQR migration similar to *egl-20* and *mab-5* (Fig 1). *bar-1(ga80* and *mu349)* displayed nearly completely-penetrant anterior PQR migration, suggesting they cause strong loss of function, although *mu349* showed a significantly weaker effect (Fig 1). *bar-1(mu63)* showed much weaker PQR defects and is likely a hypomorphic allele (Fig 1). We analyzed QL.a/p behavior in the strong *bar-1* alleles *ga80* and *mu349*. As in *egl-20* and *mab-5*, QL.a/p polarized and migrated anteriorly (Fig 10). To determine if *bar-1* resembled *egl-20* or displayed the delay of *mab-*5, we scored the percentage of migrating QL.a/p in *bar-1* mutants in which QR.a/p had begun migration at the 4.5–5.5h timepoint (Fig 10): 29% of *bar-1(ga80)* and 36% of *bar-1(mu349)* showed QL.a/p migration. This was significantly less than *egl-20* mutants (*p < 0*.*05*) and was not significantly different from *mab-5* (Fig 10). Thus, *bar-1* mutants also displayed a delay in QL.a/p anterior migration similar to *mab-5* and not seen in *egl-20*.

## Discussion

### MAB-5/Hox and EGL-20/Wnt Inhibit Anterior Migration

Here we describe the distinct behaviors of QL and QR descendants in response to Wnt signaling. After the first Q cell division, QR.a/p and QL.a/p had a rounded morphology with little or no protrusion. Shortly after division, QR.a/p began to elongate in the anterior-posterior axis by extending F-actin-rich lamellipodial protrusions to the anterior. They then began anterior migration with polarized, migratory morphology for approximately 2h, at which time they stopped migrating, become rounded, and underwent their second round of cell division. In contrast, QL.a/p remain rounded with little or no protrusive morphology and migration. Our results show that EGL-20/Wnt and MAB-5/Hox are required to prevent QL.a/p from polarizing and migrating to the anterior. When MAB-5/Hox was ectopically expressed in QR.a/p via the *mab-5(e1751)* mutation or transgenic expression of *mab-5* in both Q cells, QR.a/p polarization and migration was similarly inhibited.

### EGL-20/Wnt has MAB-5-Independent and MAB-5-Dependent Roles

**Fig 11:**
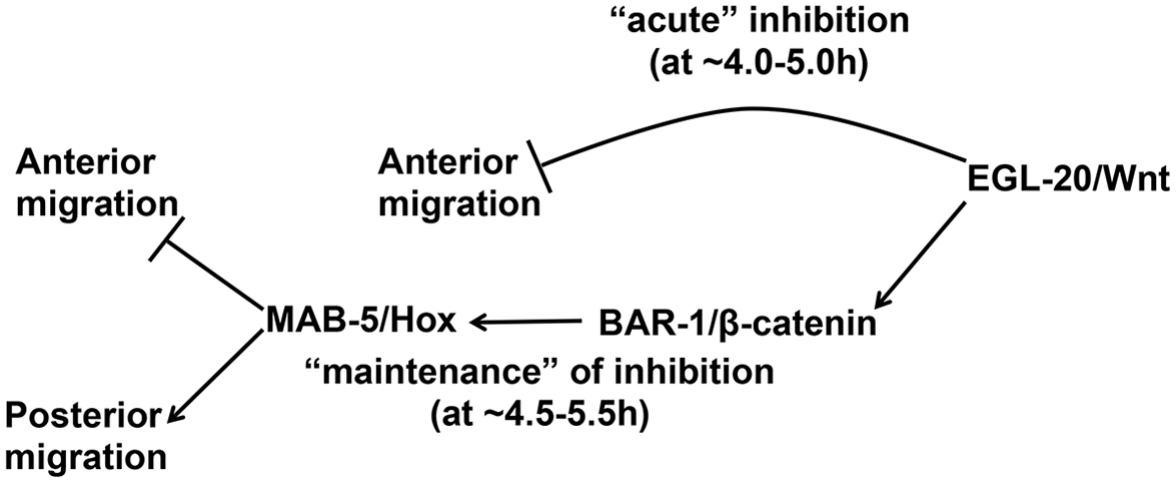
A model of EGL-20/Wnt and MAB-5/Hox function in QL migration. Data presented here indicate that both EGL-20/Wnt and MAB-5/Hox are required to inhibit anterior migration of QL.a/p. EGL-20 might immediately inhibit QL.a/p anterior migration via an “acute” mechanisms independent of BAR-1/β-catenin and MAB-5/Hox, as well as through a later “maintenance” phase that requires BAR-1 and MAB-5 and might require changes in gene expression. MAB-5 might also promote posterior migration.

Both *egl-20* and *mab-5* loss-of-function mutants displayed anterior QL.a/p migration. However, we noted a delay in anterior migration in *mab-5* not observed in *egl-20*. QL.a/p in *mab-5* remain rounded and non-migratory for a short time (~0.5-1h) before they polarized and migrated anteriorly. In contrast, QL.a/p in *egl-20* mutants resembled QR.a/p: they immediately polarized and began anterior migration. *mab-5* expression is induced in QL by canonical Wnt signaling via EGL-20/Wnt and BAR-1/ β-catenin. The delay in QL.a/p anterior migration was also observed in *bar-1/β-catenin* mutants, similar to *mab-*5. This suggests that EGL-20/Wnt has at least two distinct roles in inhibiting migration (Fig 11). EGL-20/Wnt might acutely inhibit migration in a MAB-5 and BAR-1-independent pathway, and later, via canonical Wnt signaling and MAB-5 expression, consolidate this inhibition. Consistent with this idea of step-wise regulation of Q descendant migration by EGL-20, previous studies showed that QR descendants display cell-intrinsic temporal responses to Wnt signals that determines their final positions in the anterior-posterior axis [35].

How might EGL-20 and MAB-5 inhibit QL.a/p anterior migration? EGL-20 could activate an acute response by acting directly on the ability of the cell to polarize and migrate (e.g. e.g. inhibiting cytoskeletal rearrangements and protrusive events necessary for migration). This response does not require BAR-1 or MAB-5, suggesting that it might be a non-canonical Wnt signaling pathway that acts directly on protrusive ability (e.g. the cytoskeleton). There is precedence for BAR-1-independent Wnt signaling in later QR descendant migrations, which are shortened in *Wnt* mutants independent of BAR-1/β-catenin [18]. In addition to the acute role, which is transient, EGL-20 can also activate canonical Wnt signaling via BAR-1/β-catenin resulting in MAB-5 expression. The acute EGL-20 signal might inhibit anterior migration until MAB-5 is expressed, which begins in QL during its initial migration and before division [17]. In this scenario, the delay in anterior migration in *mab-5* mutants might indicate the lag time in *mab-5* expression and regulation of target gene expression to inhibit anterior migration. Wnt signaling and *mab-5* expression remain tied in a feedback loop that ensures consistent and continuous levels of *mab-5* expression [17].

MAB-5 might consolidate inhibition of anterior migration by regulating genes involved in polarization and migration and/or genes involved in responding to an A-P guidance cue. Indeed, expression of the Hox factor LIN-39 is inhibited in QL.a/p by MAB-5 [30]. LIN-39 normally promotes anterior migration in QR.a/p by driving the expression of the transmembrane MIG-13 molecule [30]. In parallel to SDN-1/Syndecan [37], MIG-13 responds to an A-P guidance cue by mediating cytoskeletal rearrangements underlying anterior protrusion and migration [30]. Thus, MAB-5 might inhibit anterior QL.a/p migration in part by inhibiting LIN-39 expression in QL.a/p.

### MAB-5 Promotes Posterior Migration

While QL.a/p normally remain rounded and non-migratory while QR.a/p migrate anteriorly, QL.a eventually migrates posteriorly over QL.p. QL.a posterior migration is dependent on MAB-5, as we found that ectopic expression of MAB-5 in both QL and QR resulted in posterior migration of both anterior daughters. Thus, MAB-5 is not simply inhibiting migration. One possibility is that by inhibiting anterior migration, MAB-5 allows QL.a/p to respond to a later *de novo* guidance signal that directs posterior migration. By not migrating anteriorly, QL.a/p are in a position to respond to this new signal. Alternatively, MAB-5 might regulate gene expression in QL.a/p that alters response to a posterior guidance cue, possibly the same A-P cue used by QR.a/p to migrate anteriorly. As Wnts regulate Q descendant migrations, MAB-5 might alter the response of QL.a/p to Wnt signals directing anterior-posterior migrations.

The *Wnt* quadruple mutant *cwn-1; egl-20 cwn-2; lin-44* showed some posterior migration of both AQR and PQR. EGL-20/Wnt is required to activate *mab-5* expression, so it is possible that in the *Wnt* quadruple, posterior migration occurs in the absence of *mab-5*. This indicates that Wnts constitute the anterior-posterior guidance system to which *mab-5* modifies responses. In the absence of Wnts, some cells migrate posteriorly despite not having *mab-5* expression due to a disrupted anterior-posterior guidance system. It is also possible that *mab-5* is activated in an *egl-20*-independent manner in both AQR and PQR in this quadruple mutant and is responsible for posterior migration.

In sum, our results indicate that both QR.a/p and QL.a/p can both respond to an anterior migration signal. Normally, EGL-20 prevents QL.a/p from responding by first acutely inhibiting migration, likely due to the inherent sensitivity of QL to the EGL-20 signal [16]. EGL-20 also activates *mab-5* expression in QL.a/p via BAR-1/ β-catenin and canonical Wnt signaling, which likely results in changes in gene expression that maintains inhibition of anterior migration. MAB-5-induced gene expression might also then promote posterior migration. However, it is also possible that by inhibiting anterior migration, *mab-5* allows the QL.a/p cells to respond to a later posterior migration signal that is not present early in QL.a/p migration. These results indicate that *egl-20* and *mab-5* mutations do not transform QL into a QR-like fate, but rather discretely modify how these cells differentially respond to anterior-posterior guidance information.

These studies and others are beginning to paint a picture of the early migration events of the Q neuroblasts and descendants. Initial anterior QR and posterior QL migration are regulated by inherent differences in the functions of the transmembrane molecules UNC-40/DCC and PTP-3/LAR in QR versus QL [29], and does not appear to involve Wnt signaling. While the signal specifying initial anterior versus posterior migration is unknown, the Fat-like Cadherin CDH-4 non-autonomously controls UNC-40/DCC and PTP-3/LAR function in QR versus QL [26]. Due to posterior migration of QL and inherent differences in sensitivity, an EGL-20/Wnt signal acutely inhibits QL.a/p anterior migrations, possibly via a non-canonical mechanism. Via canonical Wnt signaling and BAR-1/β-catenin, EGL-20/Wnt also induces MAB-5 expression in QL.a/p, which inhibits LIN-39/Hox and thus MIG-13 expression and consolidates inhibition of anterior migration. Additionally, MAB-5 might regulate other genes that inhibit anterior migration and direct posterior migration. It will be important to identify the potential non-canonical mechanism of EGL-20/Wnt inhibition of anterior migration, and to define other genes regulated by MAB-5 to autonomously inhibit anterior migration and promote posterior migration.

## Methods

### Genetics

*C*. *elegans* were cultured by standard techniques and all experiments performed at 20°C. The following mutations and transgenes were used: LGI: *lin-44(n1792)*, *mom-5(ne12)*, *lin-17(n761)*; LGII *cwn-1(ok546)*, *ayIs9[Pegl-17*::*gfp]*, *mig-14(ga62* and *or78*); LGIII *mab-5(gk670*, *e1239*, *e2088*, *mu114*, *bx54*, and *e1751)*, *lin-39(e1760)*; LGIV *cwn-2(ok895)*, *egl-20(n585*, *ju105*, *gk453010*, *lq42*, and *lq74*); LGV *mom-2(or77)*, *lqIs58[Pgcy-32*::*cfp]*; LGX: *bar-1(ga80*, *mu63*, and *mu349);* unmapped *lqIs220[Pegl-17*::*mab-5*::*gfp]*, *lqIs221[Pegl-17*::*mab-5*::*gfp]*, *casIs330[Pegl-17*::*gfp*, *Pegl-17*::*myr*::*mCherry*, *Pegl-17*::*his-24*::*mCherry*, *Pegl-17*::*MOEabd*::*gfp]* [31]. Genotypes containing M+ indicate that homozygous animals from a heterozygous mother were scored (i.e. had wild-type maternal gene function). In the case of the *Wnt* triple and quadruple mutant, linkage group IV containing *egl-20* and *cwn-2* was balanced by the nT1 balancer chromosome marked with pharyngeal GFP.

The *Pegl-17*::*mab-5*::*gfp* transgene was produced by placing a full-length *mab-5* cDNA downstream of the *egl-17* promoter and fused in frame to the *gfp* coding region at the 3’ end (C-terminal tag). The sequence of this plasmid, pEL862, is available upon request. Six independent extrachromosomal arrays and two independent integrants (*lqIs220* and *lqIs221)* had a similar effect of causing posterior AQR migration (Fig 1).

### *egl-20* and *mab-5* Allele Sequencing

The lesions associated with *egl-20(lq42* and *lq74)* and *mab-5(mu114)* were determined by whole genome sequencing using Cloudmap [20] and confirmed with Sanger sequencing of the region. *egl-20(lq42)* was a C to T transition at chromosome IV position 9,813,864 (Wormbase release WS249) resulting in an arginine to stop; *egl-20(lq74)* was a G to A transition at 9,814,127 resulting in a cysteine to tyrosine missense; *egl-20(gk453010)* was a C to T transition at 9,814,137 resulting in an arginine to stop; and *mab-5(mu114)* was a G to A transition at chromosome III position 7,783,349 in the first exon of *mab-5* resulting in a tryptophan to stop. *mab-5(e2088)* was a complex rearrangement involving the second exon of *mab-5* at position 7,782,932. The exact molecular nature of *e2088* could not be determined.

### L1 Synchronization and Q Cell Imaging

L1 larvae were synchronized by hatching as previously described. Gravid adults and larvae were washed from plates on which many eggs had been laid. Eggs were allowed to hatch for one hour, and newly hatched larvae, all within an hour’s age of one another, were washed off and allowed to develop for specific times: 3.5h for the 3.5–4.5h timepoint; 4h for the 4-5h timepoint; 4.5h for the 4.5–5.5h timepoint; 5h for the 5-6h timepoint; 5.5h for the 5.5–6.5h timepoint; 6h for the 6-7h timepoint; 6.5h for the 6.5–7.5h timepoint; and 7h for the 7-8h timepoint. At the specified timepoint, L1 larvae were mounted for microscopic inspection and imaging of Q cell position and morphology using the *ayIs9[Pegl-17*::*gfp]* transgene.

### Scoring Q Cell Position

From micrographs of L1 larvae expressing *ayIs9[Pegl-17*::*gfp]*, the morphology and position of QL.a/p and QR.a/p were determined. QR.a/p cells were scored as migrating if they had assumed an elongated migratory morphology with anterior protrusions and/or if QL.a had separated from QL.p (Figs 2 and 3). Cells that remained rounded and adjacent to one another were scored as non-migratory. Posterior migration of QL.a in wild type and QL.a and QR.a in *mab-5* gain-of-function was defined by QX.a extending protrusions on top of QX.p, or if QX.a could not be distinguished from QX.p, indicating that QX.a was migrating behind or in front of QX.p (Figs 2 and 3). Significance of differences in Fig 4 were determined using Fisher’s exact test.

For data in Fig 10, L1 larvae were synchronized and imaged at the 4.5–5.5h timepoint, when most QR.a/p had migrated anteriorly and QL.a/p remained rounded and non-migratory. The migrations of QL.a/p were scored in those animals with migratory QR.a/p (n = 50 for each genotype). Significances of difference were determined by Fisher’s exact test.

### Time-Lapse Imaging of QL and QR

For time-lapse imaging, the *casIs330* transgene was used to visualize QL, QR and descendants. *casIs330* contains the Q-cell promoter *egl-17* driving the expression of *myr*::*mCherry* (to mark membranes), *his-24*::*mCherry* (to mark chromatin), and *MOEabd*::*gfp*, the actin-binding domain of human moesin fused to GFP, to mark F-actin [31]. Images were acquired every 2 minutes using a spinning disk confocal microscope (Zeiss Axio Observer Z1, with Yokogawa CSU-X1 Spinning Disk Unit) using previously-described methods to anesthetize and immobilize animals. Images were analyzed and assembled using ImageJ and Adobe Photoshop. Time-lapse imaging with the *casIs330* transgene delayed migrations and cell divisions by ~2-fold compared to *ayIs9* still images, but the same pattern of QL.a/p and QR.a/p migration as observed with *ayIs9* still images was conserved.

### Scoring AQR and PQR Migration

AQR and PQR are neuronal descendants of QR and QL, respectively, and migrate the longest distances of Q descendants into the head and tail of the animal. AQR normally migrates anteriorly to a region near the anterior deirid and the posterior pharyngeal bulb, and PQR normally migrates posteriorly near the phasmid ganglia posterior to the anus in the tail. AQR and PQR position was scored as described previously. The animal is divided into five regions in the anterior-posterior axis: Position 1 is the normal position of AQR; position 2 is posterior to position 1 but anterior to the vulva; position 3 is adjacent to the vulva; position 4 is the position of Q cell birth; and position 5 is the normal position of PQR posterior to the anus. AQR and PQR position was scored in 100 animals of each genotype indicated in Fig 1.

## Supporting Information

S1 Movie Division and migration of QR.a/p in wild-type. *casIs330*[*Pegl-17*::*myr*::*mCherry*, *Pegl-17*::*HIS-24*::*mCherry*, *Pegl-17*:: *MOEabd*::*GFP]* was used to image QR division and QR.a/p migrations. mCherry labeled cell membranes and chromatin (red), and GFP labeled F-actin (green). Images were acquired every 2 minutes using a spinning disk confocal microscope (Zeiss Axio Observer Z1, with Yokogawa CSU-X1 Spinning Disk Unit). Total imaging time was 200 minutes. Time-lapse imaging with *casIs330* resulted in an approximately 2-fold delay in events relative to *ayIs9* in timepoint analysis. Anterior is left, and dorsal is up.
(AVI)

S2 Movie Division and migration of QL.a/p in wild-type. *casIs330*[*Pegl-17*::*myr*::*mCherry*, *Pegl-17*::*HIS-24*::*mCherry*, *Pegl-17*:: *MOEabd*::*GFP]* was used to image QL division and QL.a/p migrations. mCherry labels cell membranes and chromatin (red) and GFP labels F-actin (green). Images were acquired every 2 minutes using a spinning disk confocal microscope (Zeiss Axio Observer Z1, with Yokogawa CSU-X1 Spinning Disk Unit). Total imaging time was 250 minutes. Time-lapse imaging with *casIs330* resulted in an approximately 2-fold delay in events relative to *ayIs9* in timepoint analysis. Anterior is left, and dorsal is up.
(AVI)
